# Seroprevalence of Herpes Simplex Viruses Types 1 and 2 in a Population, Age 15-35 Years, of Mashhad City

**DOI:** 10.52547/ibj.3828

**Published:** 2023-02-15

**Authors:** Ahmad Taherpoor, Arastoo Vojdani, Seyed Mohamad Ali Hashemi, Arian Amali, Mohammad Reza Mardani, Majid Ghayour Mobarhan, Habibollah Esmaily, Mohammad Taghi Shakeri, Mansoureh Bakhshi, Mojtaba Meshkat, Amin Hooshyar Chechaklou, Samaneh Abolbashari, Aida Gholoobi, Zahra Meshkat

**Affiliations:** 1 Department of Microbiology and Virology, Faculty of Medicine, Mashhad University of Medical Sciences, Mashhad, Iran;; 2 Student Research Committee, Faculty of Medicine, Mashhad University of Medical Sciences, Mashhad, Iran;; 3 Student Research Committee, Paramedical Department, Mashhad Medical Sciences Branch, Islamic Azad University, Mashhad, Iran;; 4 International UNESCO Center for Health-Related Basic Sciences and Human Nutrition, Department of Nutrition, School of Medicine, Mashhad University of Medical Sciences, Mashhad, Iran;; 5 Social Determinants of Health Research Center, Mashhad University of Medical Sciences, Mashhad, Iran;; 6 Antimicrobial Resistance Research Center, Mashhad University of Medical Sciences, Mashhad, Iran;; 7 Department of Community Medicine, Faculty of Medicine, Mashhad Medical Sciences, Islamic Azad University, Mashhad, Iran;; 8 Metabolic Syndrome Research Center and Medical Genetics Research Center, Mashhad University of Medical Sciences, Mashhad, Iran; † Contributed equally as first authors

**Keywords:** Herpes Simplex Virus Type 1, Herpes Simplex Virus Type 2, Iran, Seroepidemiology

## Abstract

**Background:**

Considering the high prevalence and clinical importance of herpes simplex virus (HSV) infection worldwide, we aimed to evaluate the seroprevalence of HSV-1 and HSV-2 in a population aged between 15 and 35 years in Mashhad, Iran.

**Methods:**

This cross-sectional study was conducted on 916 cases composed of 288 (31.4%) men and 628 (68.6%) women. Using ELISA method, the presence of IgM and IgG antibodies against HSV-1 and HSV-2 was assessed.

**Results:**

Among the population studied, 681 (74.3%) cases were positive for anti-HSV antibodies, while 235 (25.7%) cases were negative. Moreover, no IgMs were found and all positive subjects had IgG antibodies. Age (*p* < 0.001), occupation (*p* < 0.001), education (*p* = 0.006), smoking (*p* = 0.029), and BMI (*p* = 0.004) demonstrated a significant association with HSV-1 and HSV-2 infection.

**Conclusion:**

Our study indicates a high seroprevalence of HSV infection; however, there was no cases positive for IgM antibodies, suggesting the high prevalence of latent infection.

## Introduction

Herpes simplex viruses are double-stranded DNA viruses belonging to the Herpesviridae family^[^^1^^]^. There are two types of HSV, type 1 (HSV-1) and type 2 (HSV-2), which share numerous genomic and biological characteristics^[^^2^^]^. HSV-1 is generally transmitted via nonsexual contact during childhood, while HSV-2 is typically transmitted by sexual contact in adulthood^[^^3^^]^. Both HSV-1 and HSV-2 infections are mostly subclinical^[^^4^^]^. Following primary infection, HSV establishes latency in nerve ganglia; i.e. HSV-1 in the trigeminal ganglion, and HSV-2 in the sacral ganglion. Of note, the infection can become recurrent on a periodic basis due to virus reactivation^[^^4^^,^^5^^]^. HSV-2 infection is one of the most prevalent sexually transmitted infections^[^^6^^,^^7^^]^. It is considered as a co-factor of HIV infection^[^^8^^]^ and the main causative agent of genital herpes. Therefore, it manifests recurrent lesions in the genital and anal areas when the infection becomes symptomatic^[^^9^^,^^10^^]^. If transmission of a primary genital herpes infection occured during pregnancy, especially in the last trimester or within the giving birth, it would cause severe systematic neonatal infection^[^^11^^-^^13^^]^. HSV-1 is the primary cause of oral infection, but a number of researches have demonstrated in the last two decades that this infection has become known as the cause of genital herpes transmitted by oral-genital contact^[^^14^^]^. Blindness and encephalitis also occur because of both HSV-1 and HSV-2, though they are much less common. 

An important challenge in the management of HSV infections is the high prevalence rates of HSV-1 and HSV-2. According to the World Health Organization, as many as 3.7 billion people worldwide were infected with HSV-1, and 491.5 million showed HSV-2 infection in 2016. In a study conducted in the United States from 2005 to 2010, HSV seroprevalence was reported to be as high as 53.9% for HSV-1 and 15.7% for HSV-2^[^^15^^]^. Another study estimated the seroprevalences of HSV-1 and HSV-2 as 75.3% and 2.9%, respectively, among Jordanians^[^^16^^]^. The same high prevalence rate was found in a meta-analysis study in Iran, demonstrating that the prevalence of HSV-1, HSV-2, and total HSV infections were 42.04%, 6.5%, and 25.7%, respectively, among Iranians^[^^17^^]^. The findings of previous studies have emphasized the role of age as the most critical predictor of HSV seroprevalence^[^^18^^]^. More specifically, individuals aged 15-49 years are considered as the most important age group in terms of HSV infection^[^^19^^]^. The high risk of sexual transmission and the reproductive health outcomes indicate the value of the assessment of seroprevalence in this specific age group. As the majority of individuals who are infected with HSV are asymptomatic, unaware of their infection, or dealing with HSV infection is not prioritized by their communities; hence, surveillance of the extent of HSV infection in different countries and societies would be difficult. 

Advancements in the serological methods such as ELISA have made it feasible to measure both asymptomatic and symptomatic infections in the population and provide an epidemiologic evaluation of the burden of HSV infection^[^^20^^,^^21^^]^. Seroprevalence studies assist researchers and legislators in developing efficacious strategies for the prevention and control of the spread of this virus. Behavioral patterns such as sexual behaviors, which may lead to the transmission of HSV, can also be recognized^[^^5^^,^^8^^]^. Considering the probable association between HSV-2 and HIV infection, identifying the high-risk groupsincluding specific age groups, would help to prevent the spread of HIV^[^^5^^]^. Thus, in the current study, we aimed to evaluate the seroprevalence of HSV-1 and HSV-2 in a population aged between 15 and 35 years in Mashhad, Iran.

## MATERIALS AND METHODS

This descriptive cross-sectional study assessed 916 serum samples collected from a population of Mashhad City between 2019 and 2020. Participants with the ages of 15-35 years old were selected using a stratified random sampling method from three different regions in Mashhad. Each region was divided into nine sites, in accordance with Mashhad Healthcare Center divisions. Individuals whose age was between 15 and 35 years were identified and provided with an information brochure of the study by the local health authorities. They were then contacted by telephone calls for arranging an appointment to be visited for history taking and blood sampling. Inclusion criteria comprised men and women aged between 15 and 35 years and permanent residents of Mashhad City, as well as those who agreed to participate in the research study. Individuals under treatment for rare diseases were excluded from this study. 

Demographic information and anthropometric data were collected from all the participants after obtaining a verbal consent. Following the collection of blood samples, serum specimens were isolated and stored at -20 °C. Assessment of the samples was carried out at the Antimicrobial Resistance Research Center, Mashhad University of Medical Sciences. Anti-HSV IgG and IgM were evaluated by the ELISA method using a commercial kit (HSV-1 and -2 IgM and IgG antibody assay, Euroimmune, Germany) according to the instructions of the manufacturer. 

For statistical analysis of the data, SPSS 20 was used. Chi-squared test and independent t-test were also employed to assess the association of anti-HSV antibodies with qualitative and quantitative variables.

## RESULTS

From a total of 916 subjects, 681 (74.3%) were positive for IgG antibodies against HSV-1 and HSV-2,

though no subject was positive for anti-herpes simplex IgM. All subjects were between 15 and 35 years of age, with a mean age of 25.46 ± 5.76. Men comprised 31.4% and women 68.6% of the participants (288 and 628 individuals, respectively). The age of the cases who were positive for HSV-1 and -2 was higher than those who were negative, with a statistical significance of *p* < 0.001. Gender did not display any considerable correlation with HSV infection (*p* = 0.099). 

Concerning occupational status, our subjects were categorized into three (employed, unemployed, and student) groups; 235 (25.7%) were students, 240 (26.2%) were employed, and 441 (48.1%) were unemployed. Regarding education, 9 (1%) subjects were illiterate, 111 (12.1%) had primary school level education, and 576 (62.9%) had intermediate to secondary school level education, as well as 81 (8.8%), 117 (12.8%), and 22 (2.4%) held an associate, bachelor, and master degrees, respectively. The mean height and weight of our participants were 164.71 ± 9.52 cm and 67.24 ± 13.03 kg, respectively. The maximum and minimum BMI were 54.08 and 16.04, respectively, with a mean of 24.79 ± 4.25. Among our subjects, 22 individuals (2.4%) had hypertension, 14 (1.5%) reported diabetes, 26 (2.8%) had hyperlipidemia, and 14 people (1.5%) mentioned a history of cardiovascular disease. Also, 176 (19.2%) people stated a history of smoking, but smoking had been stopped, and 142 (15.5%) were smokers. According to our findings, occupation and education, as well as BMI and smoking criteria, exhibited a statistically significant correlation with HSV infection (*p* < 0.001, *p* = 0.006, *p* = 0.004, and *p* = 0.029, respectively); however, underlying diseases showed no remarkable association with this infection (Table 1).

## DISCUSSION

Given that most HSV infections are subclinical, evaluation of the rate of HSV prevalence has a key role in the management of the disease spread. 

The results of this study demonstrated that 74.3% of our subjects were positive for anti-HSV-1 and HSV-2 IgG, but no subject was positive for anti-HSV IgM, suggesting a high prevalence of latent infection in this population. The mean age of the cases who had anti-HSV antibodies was 26.2, which was significantly higher than those who were negative. This occurrence may be related to the increased sexual activity with the advancement of age, since sexual contact is one of the main routs of virus transmission. Furthermore, living conditions, hygiene, and culture have been pointed out as probable reasons for the association between age and HSV infection. In line with our findings, Shen et al.^[^^22^^]^ discovered a positive association between age and HSV infection. They observed an increased seroprevalence of HSV-1 from 19.2% to 95% among individuals <5 and <30 years old, respectively. Also, HSV-2 seroprevalence was 1.6% in those who were less than 30 years old and 31.2% in those with age equal or less than 60. A research conducted in Germany in 2011 determined the HSV type-specific IgG in 200 pregnant women, 1100 children aged 0 to 18 years, and 800 blood donors, and the findings revealed the correlation of age with HSV infection. That study demonstrated that the finding may be related to the age at which sexual activity begins, therefore, with the increase of age, the transmission of HSV is elevated to a great extent^[^^23^^]^.

In a study carried out in the United States, the prevalence of genital herpes was shown to be 18.6 million among 18- to 49-year-old women, accounting for two-thirds of the study population^[^^24^^]^. Another study in Finland evaluated the prevalence of HSV-1 and HSV-2 from 2003 to 2012 and revealed that 66.4% of their patients were positive for HSV-2, and 33.6% were positive for HSV-1. It was also observed that more female than male patients had HSV-1 infection, given the fact that from 2003 to 2007, 58% of the infected subjects were females, and from 2008 to 2012, the prevalence was around 63.3%^[^^25^^]^. Most HSV infections in our study were observed in women rather than men, with no statistical significance, likely since most of our subjects were females. A more balanced study population regarding gender can provide a rather accurate result. It is worth mentioning that in the study performed in Germany, the prevalence of HSV-1 antibodies in girls was lower than boys^[^^23^^]^. Regarding occupation, most of our positive cases were unemployed (51.5%), indicating the possible role of education as a requirement for most professions, since educated individuals are more likely to be hired and to follow basic hygiene principles; therefore, they are less prone to infections. This assumption is also supported by the significant correlation between education and HSV infection in our study. In a research carried out in Gilan Province of Iran, a higher prevalence of HSV-1 and -2 was observed in the less-educated individuals^[^^21^^]^. Furthermore, researchers in Saudi Arabia determined the seroprevalence of HSV and its co-infection with HIV and syphilis. They exhibited that individuals with lower education levels were more susceptible to HSV infection^[^^26^^]^. Underlying diseases did not display any statistically significant difference between the HSV positive and negative subjects. 

**Table 1:** Comparison of the characteristics between negative and positive groups for anti HSV-1 and HSV-2 IgG

Characteristics	Frequency	IgG antibodies against HSV-1 and HSV-2		*p* value
		Negative	Positive	
Age (mean ± standard deviation)		23.33 ± 5.49	26.20 ± 5.67	<0.001
				
Gender				
**Male **	288	84 (35.7)	204 (30)	0.099
**Female**	628	151 (64.3)	477 (70.0)	
				
Occupation (%)				
**Student**	235	91 (38.7)	144 (21.1)	<0.001
**Employed**	240	54 (23)	186 (27.3)	
**Unemployed**	441	90 (38.2)	351 (51.5)	
				
Education (%)				
**Illiterate**	9	3 (1.3)	6 (0.9)	0.006
**Primary school**	111	22 (9.4)	89 (13.1)	
**Intermediate to secondary school**	576	134 (57.0)	442 (64.9)	
**Associate degree**	81	26 (11.1)	55 (8.1)	
**Bach** **e** **lor degree**	117	39 (16.6)	78 (11.5)	
**Master degree**	22	11 (4.7)	11 (1.6)	
				
Currently smoking (%)				
**Yes**	142	26 (11.1)	116 (17.0)	0.029
**No**	774	209 (88.9)	565 (83.0)	
				
Hypertension (%)				
**Yes**	26	4 (1.7)	22 (3.2)	0.417
**No**	890	231 (98.3)	659 (96.8)	
				
Diabetes (%)				
**Yes**	14	2 (0.9)	12 (1.8)	0.326
**No**	902	233 (99.1)	669 (98.2)	
				
Hyperlipidemia (%)				
**Yes**	26	4 (1.7)	22 (3.2)	0.224
**No**	890	231 (98.3)	659 (96.8)	
				
History of cardiovascular diseases (%)				
**Yes**	14	1 (0.4)	13 (1.9)	0.110
**No**	902	234 (99.6)	668 (98.1)	
				
BMI (mean ± standard deviation)	916	24.1 ± 4.12	25.02 ± 4.26	0.004

However, smoking seems to be a risk factor for the spread of viral infections, likely due to the fact that the exchange of cigarettes among smoking individuals allows for HSV transmission^[^^22^^]^. Other risk factors for HSV infection have been discussed in previous investigations^[^^12^^]^. Pregnancy, for instance, is considered a risky stage for acquiring HSV infection, specifically, due to the exposure of the fetus to a possible infection and its harmful complications^[^^13^^]^. A recent study has assessed the prevalence of HSV-2 among pregnant women in Iran and highlighted that HSV-2 infection in this population has increased over the recent years^[^^11^^]^. The results of another study also indicated a direct correlation between seroprevalence of anti-HSV-2 infection in pregnant women and age^[^^27^^]^. The undifferentiated report of HSV-1 and HSV-2 seroprevalences is a limitation of our study, which makes the generalization of the results difficult. 

In conclusion, age, education, occupation, BMI, and smoking had the statistically significant association with HSV infection. The combined prevalence of HSV-1 and -2 was found to be high among our subjects, though we suggest further evaluation of HSV-1 and -2, separately. As no IgM antibodies were found in the studied population, it can be concluded that latent HSV infection is prevalent in this population. To our knowledge, this is the first study evaluating the prevalence of HSV in Mashhad. We suggest further assessment on the prevalence of HSV throughout the country, as well as redoing similar studies in the same region in the following years to determine the possible fluctuations in HSV infections. Also, determining the prevalence of viral infections would help to control the spread of such infections, as well as assist the healthcare providers to improve the efficacy of the preventive measures. 

## DECLARATIONS

### Acknowledgments

The protocol of this study was confirmed by the Ethics Committee of Mashhad University of Medical Sciences, Mashhad, Iran (ethical code: IR.MUMS.fm. REC.1396.72).

### Ethical statement

All the authors have read and approved the contents of the final manuscript and agreed to publicize this manuscript

### Data availability

 The raw data supporting the conclusions of this article are available from the authors upon reasonable request. 

### Author contributions

AT: contributed in completing the manuscript; AV: took the lead in writing the manuscript; SMAH: contributed in completing the manuscript; AA: contributed in completing the manuscript; MRM: contributed in completing the manuscript; MGM: devised the main conceptual idea; HE: contributed to the interpretation of the results; MTS: contributed to the interpretation of the results; MB: carried out the experiments; MM: contributed to the interpretation of the results; AHC: carried out the experiments; SA: carried out the experiments; AG: devised the main conceptual idea; ZM: devised the main conceptual idea. All authors provided feedback and finally proved the manuscript.

### Conflict of interest

None declared.

### Funding/support

There is no funding supported this project.
